# Early maternal mirroring predicts infant motor system activation during facial expression observation

**DOI:** 10.1038/s41598-017-12097-w

**Published:** 2017-09-15

**Authors:** Holly Rayson, James John Bonaiuto, Pier Francesco Ferrari, Lynne Murray

**Affiliations:** 1 School of Psychology and Clinical Language Sciences, University of Reading, Reading, United Kingdom; 2 Sobell Department of Motor Neuroscience and Movement Disorders, University College London, London, United Kingdom; 3 Institut des Sciences Cognitives Marc Jeannerod, CNRS/Université Claude Bernard, Lyon, France; 4 Department of Psychology, Stellenbosch University, Stellenbosch, South Africa; 5 Department of Psychology, University of Cape Town, Cape Town, South Africa

## Abstract

Processing facial expressions is an essential component of social interaction, especially for preverbal infants. In human adults and monkeys, this process involves the motor system, with a neural matching mechanism believed to couple self- and other-generated facial gestures. Here, we used electroencephalography to demonstrate recruitment of the human motor system during observation and execution of facial expressions in nine-month-old infants, implicating this system in facial expression processing from a very young age. Notably, examination of early video-recorded mother-infant interactions supported the common, but as yet untested, hypothesis that maternal mirroring of infant facial gestures is central to the development of a neural matching mechanism for these gestures. Specifically, the extent to which mothers mirrored infant facial expressions at two months postpartum predicted infant motor system activity during observation of the same expressions at nine months. This suggests that maternal mirroring strengthens mappings between visual and motor representations of facial gestures, which increases infant neural sensitivity to particularly relevant cues in the early social environment.

## Introduction

Accurate identification and analysis of facial expressions is critical for understanding others’ internal states^1^, and thus for regulating social relationships. This is particularly true for the preverbal infant, whose social world is comprised predominantly of face-to-face interactions with a primary caregiver^2^, and for whom communication is achieved largely via the ‘reading’ of faces^3^. Clearly then, it is highly advantageous for infants to detect and rapidly learn about faces very soon after birth^4^, and indeed, facial processing abilities appear at an early age. For example, even neonates demonstrate a bias towards looking at face-like stimuli^5–7^, and the ability to discriminate between different facial expressions emerges within the first few months of life^8–10^. By the end of their first year, infants can exploit information afforded by others’ expressions to guide their own behaviour in ambiguous situations^11,12^, and early difficulties in facial expression understanding have been linked to a number of adverse outcomes in later childhood^13,14^.

Processing facial expressions involves a widespread network of brain regions comprising both cortical and subcortical structures^15,16^. Essential components for socioemotional processing, including the amygdala and frontal cortex, are functional soon after birth^3,17,18^, and face-sensitive cortical areas such as the fusiform gyrus and superior temporal sulcus show some degree of facial tuning in the early months^19–21^. An extensive body of research with adults and nonhuman primates also suggests that sensorimotor brain regions, including parietal and premotor cortices, could support facial expression processing (e.g., refs^22–24^), but whether this is the case in human infants has not been investigated. Recruitment of these parietal-premotor regions while observing others’ actions is widely thought to implement a mapping from the visual representation of an action to its corresponding motor representation^25^. This ‘mirror’ or ‘action-perception matching’ mechanism is believed to play a key role in the visual processing of others’ behaviour and in regulating social interactions^26,27^. Such a mechanism may be especially important for facial expressions in the early postnatal period, allowing infants to tune their own behaviour with that of their mother during complex face-to-face exchanges^28–31^, and serving as a basis for the development of more advanced socio–cognitive skills^32^. In macaque monkeys, evidence suggests that a mechanism matching own and other facial gestures is present in the very first days of life^18,33^, but in humans, the earliest evidence comes from 30-month-old children^34^.

Our study aimed to address two important and outstanding questions concerning a facial action-perception network: i) is a mechanism coupling own and other facial expressions present in the human infant; and ii) if so, how does it develop (in particular, what is the role of the early social environment)? To answer the first question, we used electroencephalography (EEG) to measure event related desynchronization (ERD) in the mu frequency band during observation/execution of facial expressions, in a group of nine-month-old infants. One non-emotional condition (mouth opening) and two emotional conditions (happy, sad) were included, all of which are commonly occurring expressions in the infant repertoire. A scrambled control condition was also included to control for observation of any moving face-like stimulus (as in ref^34^). Mu ERD in central electrodes is a commonly used index of motor system activity, and hence of an action-perception network if seen during both action observation and performance^35–37^ (see the Supplementary Information file for more details). Note, we chose to look at mu ERD at nine months because other EEG research has already found evidence of motor system recruitment during observation of manual actions by this age (e.g., refs^38–40^).

To address the second question, we identified specific behaviours during early mother-infant interactions that could support the development of a mechanism matching own and other facial gestures. Critically, unlike *manual* actions, where self-observation during action execution could strengthen a mapping between visual and motor representations^30,41,42^ (a hypothesis supported by evidence from both infants^40^ and adults^43,44^), *facial* expressions are ‘opaque’; i.e. one cannot normally observe one’s own face while performing facial movements. Accordingly, self-observation could not facilitate the development of a facial action-perception network. Instead, development of this system may rely on maternal imitation of infant facial gestures, with caregivers acting as ‘biological mirrors’ for infants during very early interactions^29,31,41,42^. In other words, through maternal imitation (or ‘mirroring’), infants could observe the visual consequences of their own facial movements, providing the sensorimotor experience necessary to strengthen a link between motor and visual representations of facial gestures^30,41,45^.

During early mother-infant interactions, mothers regularly attempt to shape the exchange to include episodes of facial and vocal mirroring^46–48^, with the great majority of mirrors performed by the mother themselves^49,50^. This is a particularly enriching and preferred form of maternal response^51,52^, with maternal mirroring over the first nine of weeks of life found to predict the degree to which infants produce the same behaviours during subsequent social exchanges^31^. However, no previous research has investigated whether maternal mirroring guides the development of an action-perception network. Therefore, in addition to examining infant EEG responses to execution/observation of facial expressions at nine months, we also filmed the same infants interacting with their mothers at two months postpartum. These videos were coded to identify instances where mothers mirrored their infant’s facial expressions, including equivalents (smile, mouth opening, and negative) to those expressions observed during EEG acquisition later on. We chose to look at mother-infant interactions at two months because this is a privileged time in terms of face-to-face interaction and maternal mirroring of expressions, with infants showing the most interest in ‘pure’ face-to-face exchanges at this age^31,53^. We predicted that mothers’ tendency to imitate particular expressions during early interactions would relate to the strength of infant mu ERD during observation of the same expression, supporting the hypothesis that visuomotor experience provided by maternal mirroring supports the development of a facial action-perception matching mechanism.

## Results

We first present findings from the infant EEG experiment at nine months, followed by those concerning the relationship between early maternal mirroring and infant mu ERD. Video recordings of infant behaviour during EEG acquisition were examined offline, allowing trials in which infants produced facial expressions to be analysed as a separate execution condition. Details concerning EEG trial numbers and minimum requirements can be found in the Supplementary Information file. For the analysis of mu ERD during observation and execution of facial expressions, the α-level was set at 0.05 and all post-hoc tests were Bonferroni corrected. The Greenhouse-Geisser correction of degrees of freedom was used if the sphericity assumption was violated (indicated by ε). T-tests were all two-tailed.

### Nine Month EEG Experiment: Execution Trials

Before reporting on the central findings, i.e. during observation, we will describe data on execution trials to establish consistency with previous research showing a decrease in mu power during execution of facial expressions^18,33,34^ and manual actions^38–40^. This execution analysis showed a significant decrease in mu power relative to baseline in the left central electrode cluster [M = −14.40, SD = 13.04; *t*(16) = −4.55, *p* < 0.0001] and the right central cluster [M = −19.91, SD = 15.83; *t*(16) = −5.18, *p* < 0.0001]. Note, trials were collapsed across condition (i.e. happy, sad, mouth opening) for the execution analysis due to small numbers (see Supplementary Information).

**Figure 1:**
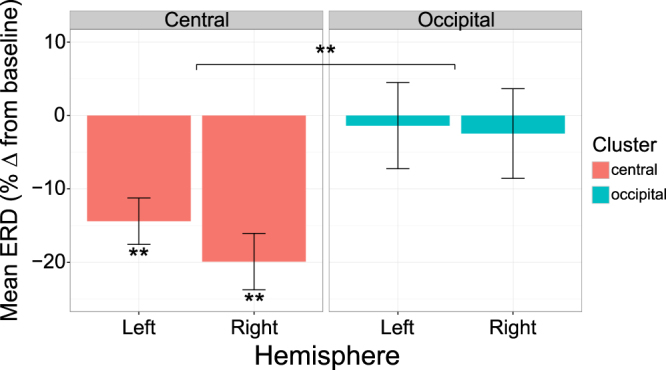
Infant mu ERD during execution. ERD during execution of facial expressions in central and occipital clusters in the left and right hemisphere. Error bars represent the mean +/− standard error, *p < 0.05, **p < 0.005.

Having confirmed that mu desynchronization (ERD) occurred during execution, a 2 × 2 repeated-measures ANOVA was conducted, with hemisphere (left/right) and electrode cluster (central/occipital) as within-subject variables (Fig. 1). The ANOVA revealed a significant main effect of electrode cluster [*F*(1, 16) = 16.96, *p* < 0.001, ${{\rm{\eta }}}_{p}^{2}$ = 0.52], with significantly greater ERD seen in central compared to occipital electrode clusters.

### Nine Month EEG Experiment: Observation Trials

**Figure 2:**
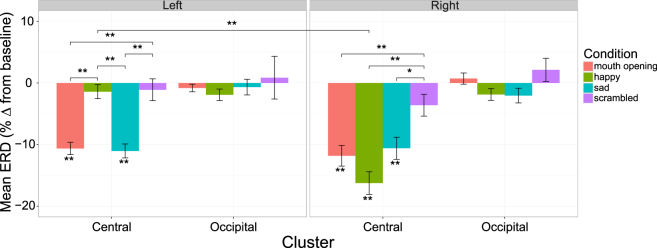
Infant mu ERD during observation. Mu ERD for each condition (mouth opening, happy, sad, and scrambled) in central and occipital clusters (left and right hemisphere). Error bars represent the mean +/− standard error, *p < 0.05, **p < 0.005.

For observation trials, a significant decrease in mu power compared to baseline was found in the left central electrode cluster for mouth opening [M = −10.63, SD = 4.40; *t*(18) = −10.54, *p* < 0.0001] and sad expressions [M = −11.04, SD = 4.95; *t*(18) = −9.73, *p* < 0.0001], but not for the happy or scrambled conditions [both *p* > 0.20]. In the right central electrode cluster, there was a significant decrease for mouth opening [M = −11.82, SD = 7.34; *t*(18) = −7.02, *p* < 0.0001], happy [M = −16.25, SD = 8.01; *t*(18) = −8.84, *p* < 0.0001], and sad [M = −10.60, SD = 7.83; *t*(18) = −5.90, *p* < 0.0001] conditions, but again, not for the scrambled condition (*p* > 0.05). There was no significant decrease in mu power relative to baseline in either occipital cluster, for any condition (all *p* > 0.05, Fig. 2).

Having established the presence of mu ERD during observation, a 2 × 2 × 4 repeated-measures ANOVA was conducted, with hemisphere, electrode cluster, and condition as within-subject variables. The ANOVA revealed a significant main effect of hemisphere [*F*(1, 18) = 5.15, *p* = 0.04, ${{\rm{\eta }}}_{p}^{2}$ = 0.22], cluster [*F*(1, 18) = 38.10, *p* < 0.0001, ${{\rm{\eta }}}_{p}^{2}$ = 0.68] and condition [*F*(3, 54) = 27.05, *p* < 0.0001 ${{\rm{\eta }}}_{p}^{2}$ = 0.60]. These results were qualified by significant hemisphere × cluster [*F*(1, 18) = 6.47, *p* = 0.02, ${{\rm{\eta }}}_{p}^{2}$ = 0.26] and significant hemisphere × condition [*F*(3, 54) = 6.61, *p* = 0.001, ${{\rm{\eta }}}_{p}^{2}$ = 0.27] interactions. A significant three-way hemisphere × cluster × condition interaction [*F*(2.06, 37.13) = 5.25, *p* = 0.009, ${{\rm{\eta }}}_{p}^{2}$ = 0.23, *ε* = 0.69] was also found, and thus two separate repeated-measures ANOVAs, for the two electrode clusters (central/occipital), were conducted to follow this up.

The analysis of central clusters (Fig. 2) revealed significant main effects of both hemisphere [*F*(1, 18) = 16.85, *p* = 0.001, ${{\rm{\eta }}}_{p}^{2}$ = 0.48] and condition [*F*(3, 54) = 15.59, *p* < 0.0001, ${{\rm{\eta }}}_{p}^{2}$ = 0.46], and a significant hemisphere × condition interaction [*F*(3, 54) = 14.07, *p* < 0.0001, ${{\rm{\eta }}}_{p}^{2}$ = 0.44]. Pairwise comparisons showed that mu ERD in the happy condition was significantly greater in the right compared to left hemisphere [*t*(18) = 6.38, *p* < 0.0001]. They also demonstrated that mu ERD in the left hemisphere was significantly greater in the mouth opening condition compared to happy [*t*(18) = −5.91, *p* < 0.0001] and scrambled [*t*(18) = −5.09, *p* < 0.0001], and significantly greater in the sad condition compared to happy [*t*(18) = −6.33, *p* < 0.0001] and scrambled [*t*(18) = −4.75, *p* < 0.005]. In the right hemisphere, there was significantly greater mu ERD in all conditions compared to scrambled [mouth opening: *t*(18) = −5.03, *p* < 0.005; happy: *t*(18) = −4.93, *p* < 0.005; sad: *t*(18) = −3.25, *p* < 0.03].

The analysis of occipital clusters (Fig. 2) revealed no significant main effects of hemisphere [*F*(1, 18) = 0.06, *p* = 0.81, ${{\rm{\eta }}}_{p}^{2}$ = 0.003] or condition [*F*(1.97, 35.41) = 2.08, *p* = 0.14, ${{\rm{\eta }}}_{p}^{2}$ = 0.10, *ε* = 0.66], and there was no significant hemisphere × condition interaction [*F*(1.51, 27.12) = 0.34, *p* = 0.65, ${{\rm{\eta }}}_{p}^{2}$ = 0.02, *ε* = 0.50]. This indicates that mu ERD was specific to central clusters and not due to changes in occipital alpha power.

### Relationship Between Maternal Mirroring at Two Months and Infant Mu Desynchronization During Observation at Nine Months

The scheme we used to code mother-infant interaction videos (devised by Murray and colleagues^31^) identifies various infant facial expressions, including smiles, mouth opening, and negative expressions. A number of maternal responses to infant expressions are also identified in the scheme, including mirroring, marking, and negative responses. More details regarding this scheme can be found in the Methods section and Supplementary Information.

Inspection of the mirroring data revealed that mothers selectively mirrored specific (smiles or mouth opening) infant expressions, rather than simply varying in their overall levels of mirroring. Accordingly, mothers were allocated to a high or low mirroring group for both smiles and mouth opening (see Methods). Negative infant expressions were rarely mirrored (two mothers only, M = 0.06, SD = 0.19), which is consistent with other research^31,54,55^, and thus were excluded from the following analyses.

A linear mixed modelling framework was used to investigate the relationship between the proportion of infant mouth opening/smiles that were mirrored (number of maternal mirroring responses normalized by the rate of infant behaviours) and mu ERD in central clusters during observation of the corresponding expression at nine months. A model with random subject-specific intercepts and hemisphere nested within subject was utilized, with condition (mouth opening/happy), hemisphere (left/right), and maternal mirroring group (mouth opening high/low; smiles high/low) for the corresponding expression (main effects and all interactions) as fixed effects. Note for all mixed models described here, visual inspection of residual plots did not reveal any deviations from homoscedasticity or normality. All *p* values were based on Kenward-Roger's corrected degrees of freedom, and all post-hoc tests (least-square means) were corrected for multiple comparisons using Tukey-Kramer contrasts.

**Figure 3:**
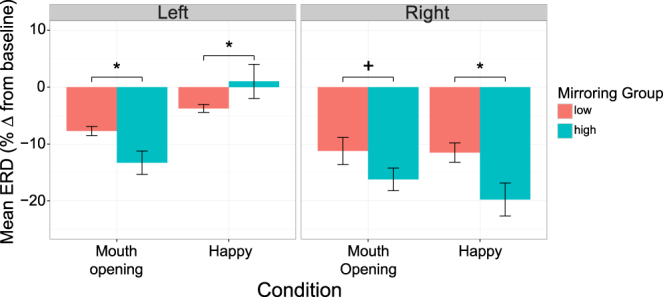
Infant mu ERD in high and low mirroring groups during observation. Infant mu ERD during observation of mouth opening and happy conditions in the low and high maternal mirroring groups for each expression, in both the left and right hemisphere. Error bars represent the mean +/− standard error, *p < 0.05, ^+^ *p* = 0.05.

Significant main effects of condition [*F*(1, 27.08) = 8.40, *p* = 0.007], hemisphere [*F*(1, 14.20) = 52.94, *p* < 0.0001], and maternal mirroring group [*F*(1, 37.94) = 5.14, *p* = 0.03] were revealed. These results were qualified by significant condition × hemisphere [*F*(1, 27.40) = 21.08, *p* < 0.0001], hemisphere × maternal group [*F*(1, 37.27) = 6.09, *p* = 0.02], and condition × hemisphere × maternal group [*F*(1, 31.10) = 7.31, *p* = 0.01] interactions. This three-way interaction was followed up with planned pairwise comparisons. In the right hemisphere, there was significantly more mu desynchronization during the happy condition in the high compared to low mirroring group [*t*(51.56) = −2.64, *p* = 0.01], and marginally so during the mouth opening condition in the high compared to low mirroring group [*t*(50.72) = −1.98, *p* = 0.053]. In the left hemisphere, there was significantly more mu desynchronization during the mouth opening condition for the high compared to low mirroring group [*t*(50.72) = −2.20, *p* = 0.03], but significantly less during the happy condition for those in the high compared to the low mirroring group [*t*(51.56) = 2.08, *p* = 0.04], likely due to the disappearance of desynchronization in the high mirroring group. See Fig. 3.

In order to confirm that effects in the previous analysis were specific to maternal mirroring, control analyses were run to rule out any influence of purely motor (infant execution) or visual experience (maternal execution) during early interactions. To do this, two linear mixed models were used to investigate whether any relationship existed between base rates of mouth opening or smiles, by infant or mother during the two month interactions, and infant mu ERD in central electrode clusters during observation of those same expressions. Both models included random subject-specific intercepts and hemisphere nested within subject, and either rate of infant execution (frequency per minute: mouth opening, M = 2.47, SD = 3.24; smiles, M = 1.82, SD = 2.13) or rate of mother expression execution (frequency per minute: mouth opening, M = 2.61, SD = 2.16; smiles, M = 4.11, SD = 1.12), along with condition, hemisphere, and their interaction as fixed effects. Neither of these analyses revealed significant main effects (both *p* > 0.15), ruling out the possibility that differences found in mu ERD during observation were due to motor experience gained from infants performing actions themselves, or to the visual experience of observing mothers performing the expressions.

Finally, we tested whether the mirroring effects above might actually be accounted for by more *general* measures of maternal behaviour, rather than the specific correspondence between infant motor activity and maternal mirroring responses. To do this, two linear models were used to investigate associations between infant mu ERD during observation and i) the overall rate of maternal mirroring (i.e. the proportion of *all* coded infant facial expressions mirrored, including those not presented in the EEG experiment), or ii) the overall prominence of maternal mirroring (i.e. the proportion of all maternal responses that comprised mirroring, to any infant expression). In each case, mu desynchronization averaged over both central electrode clusters (left/right) and unscrambled conditions (mouth opening/happy/sad) was the dependent variable. Neither of these associations was found to be significant (both *p* > 0.38). More information about these extra analyses, as well as some additional control analyses testing the specificity of these effects, can be found in the Supplementary Information file.

## Discussion

The aim of this study was to investigate whether in human infants, as in adults and macaques (e.g., refs^23,24,33^), the motor system is recruited during *observation* of facial expressions. Additionally, we wished to test the hypothesis that early maternal mirroring predicts the development of the neural mechanism mapping between self- and other-generated facial movements^30,31,41,45^. The pattern of mu ERD revealed suggests that motor regions are activated in nine-month-old infants not only during execution of facial expressions, but also during their observation, thereby supporting the existence of a facial action-perception network at this young age. In addition, and in line with our hypothesis, greater maternal mirroring of a particular facial expression (mouth opening and smiles) at two months postpartum predicted stronger infant mu ERD during observation of the same expression later on in infancy. This result constitutes the first evidence in support of visuomotor experience^29,41,42^, afforded by maternal facial mirroring, facilitating development of a neural action-perception matching mechanism for faces.

In the EEG experiment, infants showed significant mu ERD in central electrode clusters during observation of various facial expressions (mouth opening, happy, sad) relative to a static neutral face, but not during observation of scrambled versions of those same expressions. The lack of mu ERD seen during observation of scrambled stimuli indicates that desynchronization during other conditions was not simply a function of observing a moving face-like stimulus, or other attentional factors. Additionally, no significant mu ERD was found in occipital electrode clusters, suggesting that central responses were not driven by alpha desynchronization in visual cortex, and were specific to motor cortical regions. These results therefore indicate recruitment of the motor system during facial expression processing.

Of particular note was our finding that infants whose mothers mirrored either mouth opening or smiles more often during early social interactions showed greater mu ERD during observation of happy and mouth opening stimuli respectively in the later EEG experiment. Control analyses confirmed that this was not simply the result of increased motor or visual experience, and no relationship was found between more general measures of maternal mirroring and infant mu ERD. This provides evidence for maternal mirroring supporting the development of an action-perception matching mechanism by strengthening specific visuomotor mappings, rather than by broadly modulating motor system responses through some other, generalised, mechanism. One previous study with macaques does indicate a more general influence of early mother-infant interactions on the development of this mechanism^18^. Mu ERD in a group of mother-reared macaques was found to be greater during observation of facial gestures compared to nursery-reared infants, but specific experiential factors that may have contributed to this were not considered.

As well as substantiating the idea that maternal mirroring is important for development of a brain network that couples visual representations with corresponding motor programs, our results are in line with studies demonstrating a relationship between exposure to atypical emotional environments and altered infant neural activity during observation of emotional expressions^56,57^. Similarly, our findings are consistent with more recent research showing how even normal variation in mother-infant interaction quality influences infant brain development^58^, including facial expression processing^4^. Notably, however, our study extends this previous work in that it tested hypotheses concerning the role of *specific* kinds of early social experience in the development of particular neural mechanisms, rather than more generic measures of the social environment.

Although the patterns of mu ERD were very similar to those we identified in 30-month-old children^34^, one difference was revealed. Unlike the older children, who demonstrated right lateralized activity for all emotion expressions, infants in the current study exhibited *bilateral* mu ERD for sad expressions. The reason for this is unclear, but it could reflect a more refined response for happy compared to sad expressions by nine months of age. Interestingly, ERD in the happy condition also appeared more right lateralized in infants whose mothers mirrored smiles more often, consistent with right hemisphere specialization for emotional face processing^15,59^. By corollary, the lack of lateralization for sad expressions at nine months could possibly be linked to less maternal mirroring of negative infant expressions; i.e. mothers imitated negative expressions far less than mouth opening and smiles in this sample, corroborating previous research^2,31^. Given that depressed mothers show atypical levels of mirroring during early interactions^60^, responding more to negative and less to positive infant expressions^55^, it would be both clinically and scientifically relevant to explore how such differences in maternal mirroring in the context of depression might affect development of a facial action-perception mechanism; and ultimately, the later problems in affective regulation characteristic of offspring of depressed mothers.

Although our study indicates an influence of social experience in the early postpartum period, it cannot speak to the issue of the status of a facial action-perception network at birth. EEG studies with macaque infants indicate that the motor system is involved in processing facial gestures from the first days of life^33^, and that early social experience rapidly influences this system’s activation^18^, but this is not necessarily true in humans, and so far remains uninvestigated. In humans, maternal mirroring has very specific effects on emerging infant social behaviour^31^, and therefore an action-perception network could play an important part in very early face-to-face interactions. Murray *et al*.^31^ found that development of social expressiveness (e.g. production of smiles, pre-speech mouth movements such as mouth opening) was substantially affected by even brief periods of visual-motor experience (mirroring) provided by mothers over the first nine weeks of life. This could be interpreted as the infant’s brain being highly tuned to a relatively limited number of stimuli (provided by the face), and that to support social perception, the brain capitalizes on relatively infrequent periods of contingency to strengthen and refine a network that maps between own- and other-generated facial expressions^18,29,31^.

It is notable that significant mu ERD was found during observation of sad expressions in our sample when mothers rarely mirrored infants’ negative expressions. However, there are two points to keep in mind when interpreting this result. First, although mothers did not mirror negative expressions very often during the recorded interaction periods, this does not mean that they never mirrored these expressions. Potentially communicative infant expressions (such as smiles and mouth opening) are most likely to be mirrored during early interactions, but mothers also mirror expressions of negative affect^61,62^, albeit less frequently in typical populations. If we had observed more mother-infant interactions, we might have observed more instances of negative mirroring; however, we would expect the relative proportion of expressions (i.e. of negative compared to smiles and mouth opening) mirrored to remain the same^31,61,62^. If the infant brain is highly sensitive to maternal mirroring^31^, visuomotor mappings for negative facial expressions could be strengthened even with very little experience of being mirrored. Second, and as noted previously, mu ERD for sad expression observation at nine months was bilateral, whereas a right-lateralized response to this condition was found in 30-month-old children^63^. One hypothesis is that mu ERD occurs during observation of all facial expressions from a very early age in human infants, but that maternal mirroring then refines the visuomotor networks involved, resulting in right-lateralized representations for different emotional expressions at varying times.

A limitation should be acknowledged regarding the execution condition in our EEG experiment. As participants here were very young infants, it was not feasible to include an explicit condition for execution of facial expressions, which resulted in relatively few trials per expression type. The subsequent need to combine different facial expressions into one condition for analysis restricts conclusions concerning the specificity of action-perception coupling. However, the overlap in neural activity revealed during observation and execution in central regions is still indicative of an action-perception matching mechanism. Further, although these results strongly suggest that early maternal mirroring of infant facial expressions influences the degree of infant motor system activity during observation of the same expressions later on, the data presented here are correlational, and thus cannot prove causality of the relationship. Future research involving the systematic manipulation of mirroring variables would help to address this point. Finally, as with many studies in this field, our sample size was modest, and replication with a larger sample is required to establish the reliability of the effects we have reported.

In summary, our findings suggest that the motor system is recruited during observation of facial expressions in human infants, and that early maternal mirroring facilitates the development of a mechanism mapping between own- and other-generated expressions. The existence of this mechanism early on postpartum could aid in the processing of others’ facial gestures, which are exceptionally important cues for the preverbal infant, and thus in navigating the extremely complicated social world into which infants are born^18^. Given how critical this is for individual success, our results also underscore the value of analysing mother-infant dyads as tightly-coupled systems in which infant behaviour influences maternal responses, which in turn, shape development of the infant brain.

## Methods

### Participants

34 infants (19 male, 15 female) took part in the EEG experiment at nine months postpartum. Mother-infant dyads were recruited from the ‘Child Development Group’ database, maintained in the School of Psychology and Clinical Language Sciences at the University of Reading. Before analysis, a number of infants had to be excluded, leaving a final sample of 19 (age: M = 275.42 days, SD= 7.88). The infants included in the final EEG sample had all previously been recorded interacting with their mothers at two months postpartum (age: M = 63.58 days, SD = 3.75). This research was approved by the University of Reading Research Ethics Committee (31.07.14), and was conducted in accordance with the Declaration of Helsinki. Mothers gave written, informed consent before participation. Further details concerning this sample and exclusions are provided in the Supplementary Information file.

### Nine Month EEG Experiment

#### Stimuli

**Figure 4:**
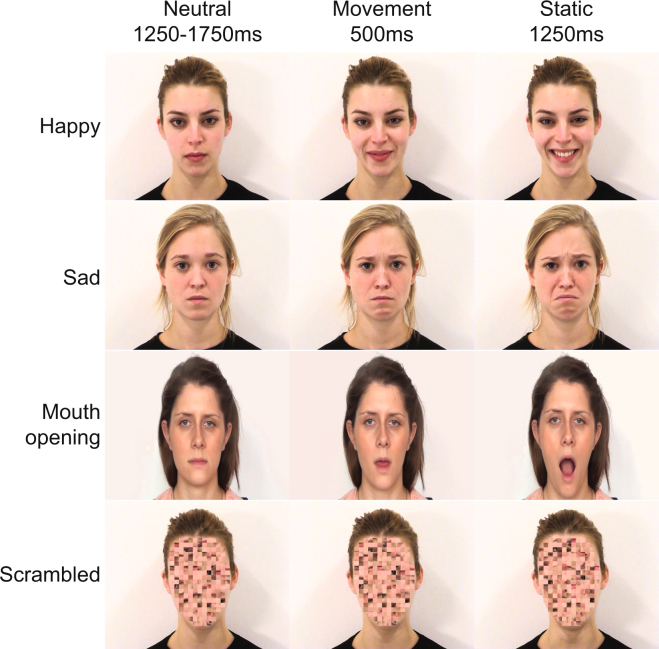
Time-course of stimuli in the four experimental conditions. Each condition included an initial, static/neutral expression, followed by a facial movement which lasted approximately 500 ms. After the movement peak, the expression was held for 1250 ms. Happy and sad stimuli were taken from the Amsterdam Dynamic Facial Expression Set^64^.

Stimuli consisted of short videos of female actors executing various facial expressions (Fig. 4). These included four experimental conditions: two emotional expressions, ‘happy’ (positive) and ‘sad’ (negative); one non-emotional expression, ‘mouth opening’; and one control condition consisting of scrambled versions of the other videos (i.e. of each happy, sad, and mouth opening video separately), whereby the face was split into a set of block regions which were randomly rearranged (see Supplementary Information). Previous studies have utilized static or non-biological moving stimuli in control conditions^24,33^; however, the scrambled stimuli condition was used here instead to control for low-level visual features and overall movement across all experimental conditions^34^. The videos featuring emotional facial expressions were taken from the Amsterdam Dynamic Facial Expression Set (ADFES), which has been well validated in previous research^64^. Equivalent videos were made for the non-emotional (mouth opening) condition, comparable with the ADFES stimuli in terms of onset, duration of movement, size, brightness, contrast, and spatial frequency. Ratings of the mouth-opening videos on a scale of −2 (negative) to +2 (positive) by a panel of 20 adults confirmed that they represented non-emotional facial expressions (M = −0.10, SD = 0.07). All videos started with a static/neutral facial expression, followed by 500 ms of movement, and 1250 ms held at the movement peak.

#### Design, procedure, and data acquisition

Infants were seated on their mother’s lap approximately 65 cm from a computer monitor. Experimental stimuli were presented on the monitor using PsychoPy v1.80.04^65^ in blocks of 4 video clips, one of each condition (i.e. one happy, sad, mouth opening, and scrambled per block; two actors per condition). These clips were randomized within blocks, and blocks themselves were pseudo-randomized so that the same condition could not be presented more than twice in succession. The inter-stimulus interval was randomized between 500 and 750 ms, and the start of the movement was randomized between 1250 and 1750 ms after the onset of the static face. The experiment was terminated if the infant became too inattentive, distressed, moved excessively, or after 25 experimental blocks had been presented.

EEG was recorded using a 128-channel Hydrocel Geodesic Sensor Net (EGI, Corp., Eugene, OR). Data were sampled at 250 Hz with an analogue band-pass filter of 0.1–100 Hz, and were recorded with the vertex as a common reference. Impedances were kept below 50 kΩ. An experimental block began when triggered manually by an experimenter who was watching the participant on a screen from another section of the room. Trial blocks were triggered as soon as the infant was attentive to the monitor. Synchronous video recordings of the experiment were examined offline to allow exclusion of trials in which the infant was inattentive, and to enable coding of infant expression execution.

#### Behavioural coding

To identify trials in which infants executed the facial expressions presented during experimental blocks, their expressions (happy, sad, and mouth opening) were coded offline from the video recordings. All videos were coded by a research assistant blind to the experimental condition being presented. Videos were viewed in real-time and frame-by-frame to accurately identify onsets and offsets of movements. A second independent researcher coded a random 20% of the videos to establish inter-rater reliability, with very good reliability obtained (time-unit *ĸ* = 0.85-0.86, event *ĸ*=0.92).

#### Data pre-processing and analysis

After viewing the video recordings of infants during the experiment and marking periods of inattention using EGI software (NetStation v4.3.1; Electrical Geodesics, Inc., Eugene, OR), EEG data were exported and analysed using the EEGLAB v13.3.2 toolbox^66^. More details about the pre-processing steps used before data analysis can be found in the Supplementary Information.

To compare power relative to baseline in the mu band, event related spectrums (ERSs) were computed for each condition using built-in EEGLAB procedures. Time-frequency decompositions were computed with a fast Fourier transform using a 1-second Hanning window with 50% overlap in 1 Hz bins from 2–35 Hz. To make results comparable with those of other studies, log spectral power was converted to absolute power, and averaged across the 6–9 Hz bins (corresponding to the mu range typically used in research with infants at this age, e.g., refs^39,40,67^). For observation, event-related desynchronization (ERD)^68^ was then computed as the percentage change of the average absolute power over a 0–750 ms time window (from the onset of facial movement in experimental stimuli until 250 ms after the peak of the full expression) from the condition-specific baseline averaged over −650 ms to −50 ms (prior to the onset of the observed facial movement). For execution, the same method of ERD calculation was used, but the 750 ms time window analysed began 250 ms before the onset of the infant’s own movement to 500 ms after^35,68–70^. The baseline used for execution was −1050 ms to −300 ms prior to the infant’s movement onset.

**Figure 5:**
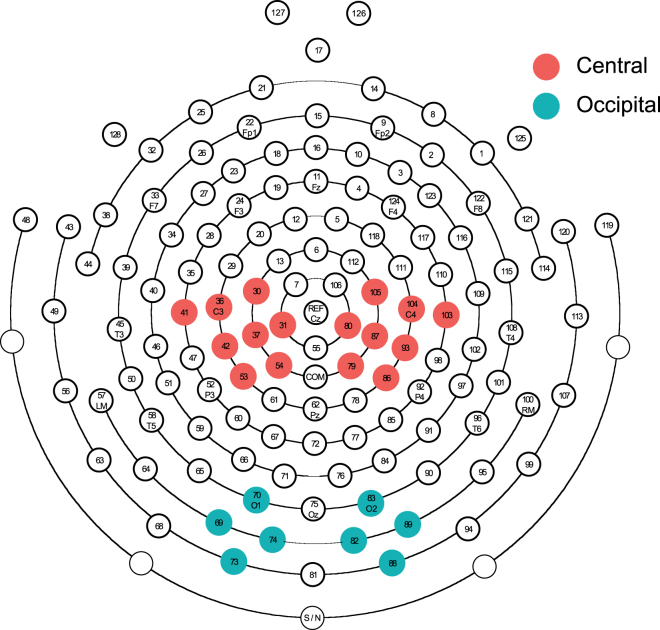
Electrode clusters used for analysis of mu ERD. Location of electrodes included in the left/right central clusters (red) and left/right occipital clusters (blue).

ERD was calculated for four clusters of electrodes. These were comprised of two central clusters (left and right hemisphere) located around standard C3 and C4 sites for mu rhythm recording, and two occipital clusters (left and right hemisphere) located around standard O1 and O2 sites to control for visual alpha responses^71^ (Fig. 5). For each cluster, in each experimental condition, the ERD values were calculated for each participant.

### Two Month Mother-Infant Interactions

#### Design, procedure, and coding

All infants who took part in EEG experiment at nine months of age had been visited at home by a researcher at two months postpartum. During this visit, mothers were asked to interact face-to-face with their infant for three minutes. For this interaction period, infants were placed on a semi-reclined changing mat, and mothers were seated opposite them on the floor. A camera was positioned to capture a full-on view of the infant’s face, as well as a side-view of the mother. Two mirrors were also utilized, one behind infants to capture a full-on view of the mother’s face, and one next to the infant in case they turned away from the camera.

We used a scheme devised by Murray *et al*.^31^ to identify instances of maternal mirroring of infant facial expressions from the mother-infant interaction videos. The scheme identifies infant and maternal events that take place during early social interactions, based on the literature concerning early infant social development and early mother-infant engagement^46,47^. Infant facial expressions coded in this scheme include an equivalent of each expression included in the nine month EEG experiment (mouth opening, happy, sad). A number of maternal responses to infant behaviours are also identified in this scheme, including maternal mirroring. ‘Mirroring’ here is defined as a maternal response that is an exact match of the infant’s behaviour, or a match of the main features with some minor modification; this could be, for example, an additional feature added to a direct match (e.g., a vocalisation to a clear mouth opening), or the omission of some element (e.g., mirroring the facial expression of a cry but without sound). See Supplementary Information file and Murray *et al*.^31^ for more details.

All interaction videos were coded by a research assistant blind to experimental hypotheses. A second independent researcher coded a random 20% of the EEG videos to establish inter-rater reliability, with very good reliability scores obtained (infant events *κ* = 0.90; maternal events *κ* = 0.83).

#### Linking the interaction and EEG data

In Table 1, descriptive statistics are given concerning the proportion of infant facial expressions mirrored overall (number of maternal mirroring responses to any infant facial expression/number of infant facial expressions performed), the total proportion of maternal responses to infant facial expressions that were mirroring (number of mirroring responses to any infant facial expression/number of maternal responses of any kind to infant facial expressions), and the proportion of expressions included in the nine month EEG experiment that were mirrored by mothers (mouth opening, smiles, negative expressions: number of maternal mirroring responses to a specific expression/number of times infant produced that specific expression). Additional details regarding infant facial movements and maternal mirroring of these can be found in the Supplementary Information and Supplementary Table S1.

**Table 1:** Means and standard deviations (SD) for maternal mirroring measures.

	Mean (SD)
Percentage of all infant facial expressions mirrored	14.86% (11.99)
Percentage of mirroring responses (out of all response types) to infant facial expressions	73.35% (29.72)
Percentage of mouth openings mirrored	27.58% (24.24)
Percentage of smiles mirrored	55.49% (37.98)
Percentage of negative expressions mirrored	6.29% (19.27)

Examination of responses to the different facial expressions revealed clear bi-modal distributions in each case, with mothers falling naturally into one of two groups in terms of how often they mirrored infant mouth opening and smiles. Only two mothers ever mirrored an infant’s negative expression, so this behaviour was not considered when grouping the mothers or in the analyses of specific expressions. Therefore, based on how often mothers mirrored mouth opening and smiles, dyads were split separately into two groups for analysis of how mirroring smiles and mouth opening related specifically to infant mu ERD during observation of these expressions later on (see Supplementary Information and Supplementary Fig. S2). In accordance, these groups were labelled as ‘low mirroring’ or ‘high mirroring’. For mouth opening, there were 8 dyads included in the low group and 7 in the high; and for smiles, 10 in the low and 6 in the high mirroring group. Considering the distribution of data points and the novel nature of this study, this approach was deemed the most appropriate here. Indeed, this type of group division is employed very widely in the literature concerning links between maternal responses during early interactions and infant development^52,60,72^. These include studies about how often mothers mirror their infants during early social exchanges, where typically mothers have been found to fall into high and low groups (e.g., refs^48,60,72^), and in the monkey literature (e.g., refs^28,73^), where such divisions are often used due to the nature of data available (e.g., to divide infant imitators and non-imitators of facial gestures for comparison).

### Data availability

Further details can be found in the Supplementary Information and any other relevant data are available from the corresponding author on reasonable request.

## Electronic supplementary material

Supplementary Information
