# Supplementary material for: Early maternal mirroring predicts infant motor system activation during facial expression observation
Source: Sci Rep. 2017 Sep 15;7:11738. doi: 10.1038/s41598-017-12097-w (PMC5601467; doi:10.1038/s41598-017-12097-w)
Supplement: Supplementary file 1 — Supplementary Information [file 41598_2017_12097_MOESM1_ESM.pdf]

Early maternal mirroring predicts infant motor system activation during facial expression observation

Holly Rayson[1*], James John Bonaiuto[2], Pier Francesco Ferrari[3], Lynne Murray[1,4,5]

[1] School of Psychology and Clinical Language Sciences, University of Reading, Reading, United Kingdom

[2]Sobell Department for Motor Neuroscience and Movement Disorders, University College London, United Kingdom

[3]Institut des Sciences Cognitives Marc Jeannerod, CNRS/Université Claude Bernard Lyon, France

[4]Department of Psychology, Stellenbosch University, South Africa

[5]Department of Psychology, University of Cape Town, South Africa

***Corresponding Author:** Holly Rayson

Email: holly.rayson@reading.ac.uk

Telephone: +44 (0)781 737 2614

Fax: +44 (0)118 378 6715

Address: School of Psychology and Clinical Language Sciences, University of Reading, Earley Gate, Whiteknights Road, Reading, RG6 7AL, United Kingdom

SUPPLEMENTARY INFORMATION

PARTICIPANTS

Of the 34 infants who took part in the EEG experiment at nine months postpartum, nine were excluded before analysis due to fussiness during net placement/the experiment (N = 7) or technical problems (N = 2). Infants were also required to have a minimum of five usable observation trials per condition after data pre-processing, leaving a final sample of 19 (11 male, 8 female). This minimum trial requirement is in keeping with similar infant mu rhythm studies [e.g. ,1–4], as is the rate of data loss and sample size.

Mother-infant dyads were recruited when infants were aged 4-6 weeks, and were recorded interacting face-to-face in mothers' homes at two months postpartum. All infants in the final sample were born healthy, full term (i.e. 37-42 weeks), and of a normal birthweight (M = 7lb 14oz, SD = 0.03). The average age of mothers at the time of birth was 30.89 years (SD = 4.85). All mothers had been educated to at least a GCSE level, 95% identified as 'White (British/Irish/other White background)', and 95% were either married or cohabiting.

EEG STIMULI

As in Rayson et al. [4], the scrambled versions of each video were produced by dividing the face region into square blocks (18 × 18 pixels), randomly shuffling these blocks in the first frame of the video, and then applying the same transformation to each subsequent frame. This resulted in a video with similar low-level visual and motion features as the original, but with an unrecognizable movement.

MU DESYNCRONIZATION AS AN INDEX OF MOTOR AND ACTION-PERCEPTION SYSTEM ACTIVITY

In EEG studies, mu rhythm event related desynchronization (ERD) is used as an index of motor system activity, defined as a reduction in mu power in central electrodes [5]. Mu ERD is associated with voluntary movement, but is considered a good proxy measure of action-perception system activity [5–8] as it actually occurs during both the observation and execution of actions [e.g. ,1,9], is likely generated in the sensorimotor cortex [10,11], and during the observation of others' actions, co-varies with BOLD activity in classic action-perception regions [12]. As a non-invasive technique, EEG is the most practicable and frequently utilized method to investigate motor system activity in the developing brain [e.g. ,1–3].

PRE-PROCESSING OF NINE MONTH EEG DATA

The PREP pipeline toolbox was used to identify and remove noisy channels [13]. Data were then bandpass filtered at 2-35 Hz. For analysis of observation trials, epochs ranging from 1s before the

onset of the adult facial expression to 2s after the start of the movement were extracted, and those containing any infant execution of facial expressions (identified in behavioural coding of EEG videos) were discarded. Execution trials were extracted by epoching the data from 1.5s prior to the onset of infant expressions to 2s after. Epochs that contained previously marked periods of inattention and epochs in which more than 15% of channels exceeded +/- 250µV were excluded. A natural-gradient logistic infomax independent component analysis (ICA) was performed on the data (the runica algorithm [14]) to decompose the EEG mixed signals into their underlying neural and artefactual components (such as eye and muscle movements). Artefact components were identified and removed using the ADJUST algorithm (v1.1 [15]), as well as by applying a rejection threshold to the entropy of the activity of each component over all trials (8580), the kurtosis of the activity (82), as well as the kurtosis of the component's spatial map (9.8 [16]). Any missing channels were interpolated using spherical interpolation and average re-referencing was applied.

ANALYSIS OF NINE MONTH EEG DATA

Observation trials

A minimum of five trials per observation condition was required for infants to be included in the analysis, in keeping with other investigations of the mu rhythm during early infancy. This left a total of 19 participants with an average of 34.21 trials (SD = 13.66) overall per participant (mouth opening, M = 8.63, SD = 3.93; happy, M = 8.05, SD = 3.64; sad, M = 8.84, SD = 3.24; scrambled, M = 8.68, SD = 4.14).

Before comparing them to each other, mu power during each condition (mouth opening/happy/sad/scrambled), in each electrode cluster (central/occipital) and hemisphere (left/right), was compared to the baseline period. This was done in order to confirm mu *desynchronization* (a reduction in power compared to baseline) took place, rather than just mu *suppression* (a reduction in power in one condition compared to another, but not necessarily compared to baseline). This is recommended as best practice for infant mu rhythm studies [5].

Execution trials

There were not enough instances of each expression to analyse separately; therefore execution was collapsed across expression type (mouth opening/happy/sad). This left 17 participants with a minimum of three execution trials each (M = 8.94, SD = 4.63), in accordance with the minimum execution trials specified in similar infant EEG studies [e.g. ,2,3,17].

Again, to check whether desynchronization occurred rather than mere suppression, changes in mu power compared to baseline were examined for combined execution conditions in central and occipital electrode clusters in the left and right hemisphere.

Note, when including only those infants with five or more execution trials (n = 14) in the same analysis, results remained consistent (i.e. there was still significant mu desynchronization in left and right central clusters compared to baseline (both $p < 0.005$), and there was significantly more desynchronization in central compared to occipital clusters ($p < 0.006$; Supplementary Fig. S1).

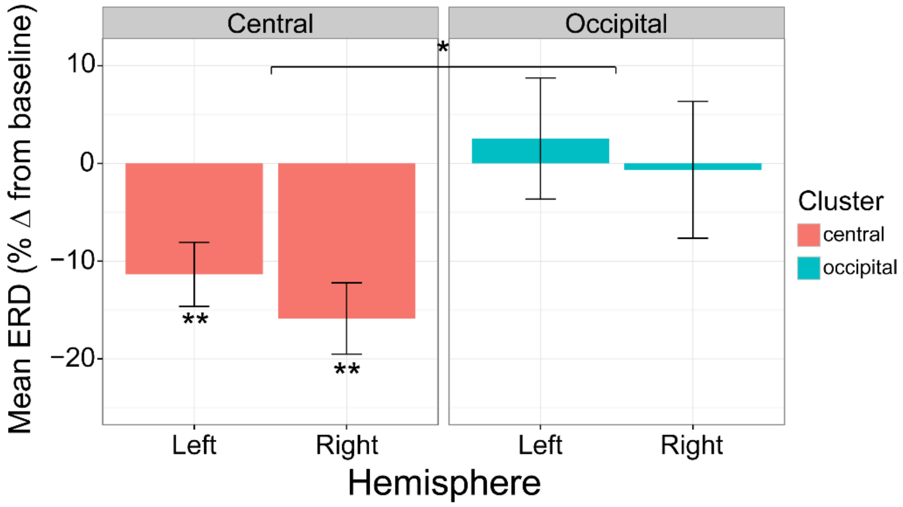

Supplementary Figure S1: Infant mu ERD during execution with five trials required.
ERD during execution of facial expressions in central and occipital clusters in the left and right hemisphere. Error bars represent the mean +/− standard error, * p < 0.05, ** p < 0.005.

TWO MONTH MOTHER-INFANT INTERACTIONS

Coding Scheme

Using the scheme devised by Murray and colleagues [18], videos were coded on a one second time base using purpose built software for identifying associations between maternal and infant behaviours. A number of infant behaviours are identified in the scheme, including facial expressions, vocalizations, and biological events. The facial movements included are as follows: tongue protrusion, mouth opening, active movements of the lips and tongue, raised brow, smiles, non-social mouth movements (e.g., chewing or sucking), and negative expressions (cry face, negative mouth, and negative eyes). Identified behaviours are mutually exclusive, representing distinct, discrete events with clear onsets.

As well as mirroring, other maternal responses were identified using this scheme. These include 'marking', which is an affirming response to an infant behaviour, but that differs from the behaviour in form and involves 'attention-attracting' cues; and 'negative' responses, which are rejecting or misattuned in terms of valence or intensity. At the time postpartum this scheme is aimed at (up to nine weeks), infants do not detect events as contingent if they occur more than three seconds after their own behaviour [19]. Additionally, mothers almost exclusively respond to infants within two seconds of infant expression [20], therefore maternal responses were only coded as contingent if they occurred within two seconds of the infant's behaviour. See Murray et al. [18] for more coding scheme details.

ANALYSIS OF THE RELATIONSHIP BETWEEN TWO MONTH INTERACTIONS AND NINE MONTH EEG

Splitting dyads into high and low mirroring groups

Below, the proportions of infant expressions mirrored by mothers that correspond to the stimuli used in the nine month EEG experiment (happy and mouth opening) are presented. Mothers were split into 'low' and 'high' mirroring groups based on natural splits in the data for smiles and mouth opening. This resulted in 10 mothers being included in the low and 6 in the high group for smiles, and 8 in the low and 7 in the high group for mouth opening. Note, only two mothers mirrored negative expressions so this was not looked at further, and numbers included in the mirroring groups are less than the number of infants with usable EEG (N = 19) as not all infants performed mouth opening or smiles during the interaction period recorded. Infants needed to have performed at least one of these expressions for the proportion of mirroring to be calculated and to be included in the analysis. See Supplementary Fig. S2

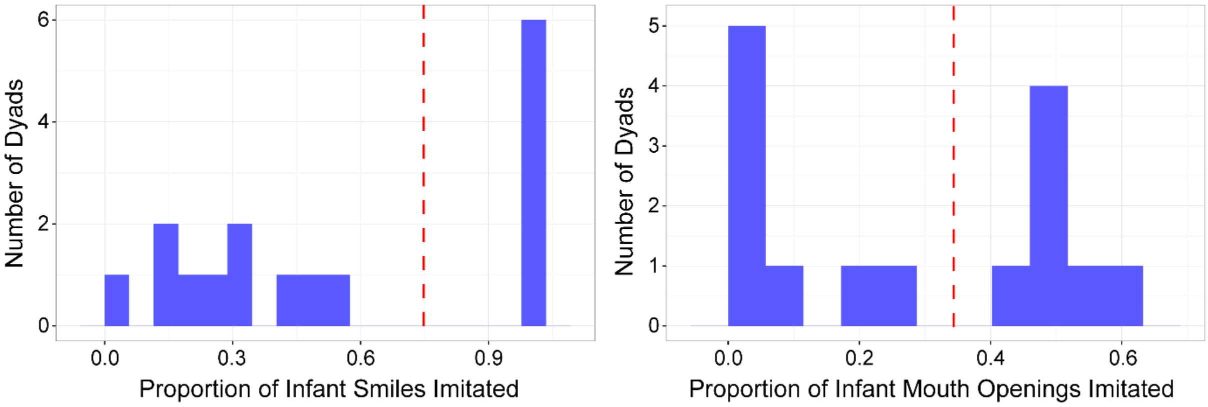

Supplementary Figure S2: Proportion of infant expressions mirrored by mothers during face-to-face interactions at two months

The histogram on the left shows the proportions mothers mirrored infant smiles, and the histogram on the right shows the proportions mothers mirrored mouth opening. The dotted line represents the point at which dyads were split into high and low mirroring groups.

Frequency of infant facial movements and maternal mirroring during early interactions

Supplementary Table S1 summarises the amount that infants here performed the expressions identified by the Murray et al. [18] coding scheme, as well as the percentage of these expressions mirrored by mothers. The expressions included in the EEG experiment stimuli at nine months were the ones (apart from non-social movements) produced by most infants during early interactions, with mouth opening and smiles mirrored most often by mothers. These findings are in accordance with previous research demonstrating that mothers tend to mirror facial movements that can be perceived as communicative [18,21–23], especially smiles and pre-speech mouth movements such as mouth opening [18], and with studies concerning the overall frequency of maternal responses to infant behaviours during early interactions [18,24].

Supplementary Table S1: Infant execution of expressions and the percentage mirrored by mothers at two months postpartum

**Infant Expression**	**Mean Frequency Per Minute (SD)**	**Mean Percentage Mirrored (SD)**
Non-social mouth movement	4.04 (1.97)	1.50% (3.08)
Tongue protrusion	0.21 (0.32)	0.0% (0.0)
Mouth opening	2.47 (3.24)	27.58% (24.24)
Yawn	0.11 (0.19)	0.0% (0.0)
Active lips / tongue movement	0.0 (0.0)	N.D.
Smile	1.82 (2.13)	55.49% (37.98)
Raised brow	0.19 (0.28)	4.17% (11.78)
Negative mouth	0.23 (0.35)	14.29% (37.80)
Negative eyes	1.60 (2.29)	1.81% (6.03)
Cry face	0.93 (2.23)	11.11% (27.22)

Control analyses testing for specificity of mirroring effects

The relationship between mu ERD in central clusters (averaged over hemisphere and mouth opening/happy/sad conditions) and the proportion of infant facial expressions mirrored overall (number of maternal mirroring responses to any infant facial expression/number of infant facial expressions performed) was explored as well as the total proportion of maternal responses to infant facial expressions that were mirroring (number of mirroring responses to any infant facial expression/number of maternal responses of any kind to infant facial expressions). These analyses were conducted to exclude more general effects of maternal mirroring, which would not support a role in strengthening visuomotor mappings for particular expressions. Again, proportions were used instead of the raw frequencies to account for the fact that infants may have produced different amounts of facial expressions, and that mothers may have made a different number of responses.

Additional control analyses

We conducted a number of additional control analyses which further support conclusions made in the main manuscript regarding specificity of the relationship between early maternal mirroring and infant mu ERD. To confirm that the effects of early maternal mirroring were specific to central electrode clusters, a linear mixed model was used to investigate the relationship between the proportion of infant mouth opening/smiles that were mirrored and mu ERD in occipital clusters during observation of the corresponding expression. A model with random subject-specific intercepts and hemisphere nested within subject was utilized, with condition (mouth opening/happy), hemisphere (left/right), and maternal mirroring group (mouth opening high/low; smiles high/low) for the corresponding expression (main effects and all interactions) as fixed effects. This revealed no significant main effects or interactions (all $p > 0.05$). Furthermore, we used two linear mixed models to exclude any influence on occipital clusters of purely motor (infant execution) or visual experience (maternal execution) during early interactions. Both models included random subject-specific intercepts and hemisphere nested within subject, and either rate of infant execution (mouth opening/smiles) or rate of mother expression execution (mouth opening/smiles), along with condition, hemisphere, and their interaction as fixed effects. Again, no significant main effects or interactions were revealed (all $p > 0.08$).

We also examined the relationship between maternal mirroring and infant mu ERD during execution (combined mouth opening/happy/sad) of facial expressions. Two linear models were used to look at mirroring of mouth opening and smiles separately, with random subject-specific intercepts utilized,

and hemisphere and maternal mirroring group (main effects and all interactions) as fixed effects. No significant main effects or interactions were revealed (all $p > 0.7$). In addition, we looked at ERD averaged over hemisphere and performed two t-tests to see if there was a difference between low and high mirroring groups for smiles or mouth opening groups during execution. Neither of these were found to be significant (smiles, $p > 0.6$; mouth opening, $p > 0.4$).This suggests that differences during observation were due to differences in the strength of visuomotor mapping, rather than variation in pure motor system development.

We used two linear models (one for smiles and one for mouth opening mirroring) to explore whether mirroring group ERD differences were specific to the same action, with random subject-specific intercepts and hemisphere nested within subject, and maternal mirroring group (high/low) of smiles or mouth opening, observation condition (happy/mouth opening/sad/scrambled), and hemisphere (main effects and interactions) as fixed effects. For smiles, there was no significant interaction between imitation level and condition ($p > 0.2$). For mouth opening, there was a significant interaction between imitation level and condition [$F(3, 78) = 21.44$, $p < 0.01$], however pairwise comparisons (corrected for multiple comparisons using Tukey-Kramer contrasts) revealed that there was only a significant difference between low and high mirroring groups for mouth opening ERD ($p < 0.05$). These results suggest that the effect of maternal mirroring on infant ERD during facial expression observation was specific to the mirrored action, which provides additional support for early mirroring strengthening the mapping between visual and motor representations, rather than having a more general influence.

Finally, we conducted two linear mixed models to examine the relationship between mu ERD during different observation conditions (happy/sad/mouth opening/scrambled) and the proportion of infant expressions (overall) that were mirrored, and the proportion of maternal responses (to any infant expression) that were mirroring. These models included random subject-specific intercepts, with the maternal mirroring variable (proportion of infant expressions mirrored or proportion of maternal responses that were mirroring) and observation condition (main effects and interactions) as fixed effects. These revealed no significant main effect of more general measures of maternal mirroring, nor an interaction between these measures and observation condition. These results further support the hypothesis that early maternal mirroring provides infants with the visual feedback necessary to influence development of a neural matching mechanism, by ruling out the possibility that the differences in infant mu ERD between the high/low mouth opening and smile maternal mirroring groups were due to more general measures of maternal mirroring.

REFERENCES

1. Cannon, E. *et al.* Relations between infants' emerging reach-grasp competence and event-related desynchronization in EEG. *Dev. Sci.* **19,** 50–62 (2016).

2. Marshall, P. J., Young, T. & Meltzoff, A. Neural correlates of action observation and execution in 14-month-old infants: an event-related EEG desynchronization study. *Dev. Sci.* **14,** 474–80 (2011).

3. Saby, J. N., Marshall, P. J. & Meltzoff, A. Neural correlates of being imitated: an EEG study in preverbal infants. *Soc. Neurosci.* **7,** 650–61 (2012).

4. Rayson, H., Bonaiuto, J., Ferrari, P. & Murray, L. Mu desynchronization during observation and execution of facial expressions in 30-month-old children. *Dev. Cogn. Neurosci.* **19,** (2016).

5. Cuevas, K., Cannon, E., Yoo, K. H. & Fox, N. A. The Infant EEG Mu Rhythm: Methodological Considerations and Best Practices. *Dev. Rev.* **34,** 26–43 (2014).

6. Pineda, J. A. The functional significance of mu rhythms: translating 'seeing' and 'hearing' into 'doing'. *Brain Res. Brain Res. Rev.* **50,** 57–68 (2005).

7. Vanderwert, R. E., Fox, N. A. & Ferrari, P. The mirror mechanism and mu rhythm in social development. *Neurosci. Lett.* **540,** 15–20 (2013).

8. Fox, N. A. *et al.* Assessing human mirror activity with EEG mu rhythm: A meta-analysis. *Psychol. Bull.* **142,** 291–313 (2016).

9. Lepage, J.-F. & Théoret, H. EEG evidence for the presence of an action observation-execution matching system in children. *Eur. J. Neurosci.* **23,** 2505–10 (2006).

10. Hari, R., Salmelin, R., Mäkelä, J. P., Salenius, S. & Helle, M. Magnetoencephalographic cortical rhythms. *Int. J. Psychophysiol.* **26,** 51–62 (1997).

11. Denis, D., Rowe, R., Williams, A. M. & Milne, E. The role of cortical sensorimotor oscillations in action anticipation. *Neuroimage* **146,** 1102–1114 (2017).

12. Arnstein, D., Cui, F., Keysers, C., Maurits, N. M. & Gazzola, V. μ-suppression during action observation and execution correlates with BOLD in dorsal premotor, inferior parietal, and SI cortices. *J. Neurosci.* **31,** 14243–9 (2011).

13. Bigdely-Shamlo, N., Mullen, T., Kothe, C., Su, K.-M. & Robbins, K. A. The PREP pipeline: standardized preprocessing for large-scale EEG analysis. *Front. Neuroinform.* **9,** 16 (2015).

14. Delorme, A. & Makeig, S. EEGLAB: an open source toolbox for analysis of single-trial EEG

dynamics including independent component analysis. *J. Neurosci. Methods* **134,** 9–21 (2004).

15. Mognon, A., Jovicich, J., Bruzzone, L. & Buiatti, M. ADJUST: An automatic EEG artifact detector based on the joint use of spatial and temporal features. *Psychophysiology* **48,** 229–40 (2011).

16. Delorme, A., Makeig, S. & Sejnowski, T. Automatic Artifact Rejection For EEG Data Using High-Order Statistics And Independent Component Analysis. *Proc. 3rd Int. Work. ICA* **457,** 462 (2001).

17. Gerson, S. A., Bekkering, H. & Hunnius, S. Short-term Motor Training, but Not Observational Training, Alters Neurocognitive Mechanisms of Action Processing in Infancy. *J. Cogn. Neurosci.* **27,** 1207–14 (2015).

18. Murray, L. *et al.* The functional architecture of mother-infant communication, and the development of infant social expressiveness in the first two months. *Sci. Rep.* **6,** 39019 (2016).

19. Gergely, G. & Watson, J. S. *Early socio–emotional development: Contingency perception and the social-biofeedback model.* (Lawrence Erlbaum Associates Publishers, 1999).

20. Nicely, P., Tamis-LeMonda, C. S. & Bornstein, M. H. Mothers' attuned responses to infant affect expressivity promote earlier achievement of language milestones. *Infant Behav. Dev.* **22,** 557–568 (1999).

21. Pawlby, S. in *Studies in mother–infant interaction* (ed. Shaffer, H.) 203–224 (Academic Press, 1977).

22. Moran, G., Krupka, A., Tutton, A. & Symons, D. Patterns of maternal and infant imitation during play. *Infant Behav. Dev.* (1987). doi:10.1016/0163-6383(87)90044-0

23. Malatesta, C. Z., Culver, C., Tesman, J. R. & Shepard, B. The development of emotion expression during the first two years of life. *Monogr. Soc. Res. Child Dev.* (1989).

24. Malatesta, C. Z. & Haviland, J. M. Learning display rules: the socialization of emotion expression in infancy. *Child Dev.* **53,** 991–1003 (1982).
